# Anthropometric and Motor Competence Classifiers of Swimming Ability in Preschool Children—A Pilot Study

**DOI:** 10.3390/ijerph17176331

**Published:** 2020-08-31

**Authors:** Ilir Gllareva, Nebojša Trajković, Draženka Mačak, Tijana Šćepanović, Anja Kostić Zobenica, Aleksandar Pajić, Besim Halilaj, Florim Gallopeni, Dejan M. Madić

**Affiliations:** 1 Faculty of Physical Education and Sport, Prishtina University, 10000 Pristina, Kosovo; i.gllareva@gmail.com (I.G.); besimhalilaj@yahoo.com (B.H.); 2 Faculty of Sport and Physical Education, University of Novi Sad, 21000 Novi Sad, Serbia; nele_trajce@yahoo.com (N.T.); tijanascepanovic021@gmail.com (T.Š.); dekimadic@gmail.com (D.M.M.); 3 Faculty of Technical Sciences, University of Novi Sad, 21000 Novi Sad, Serbia; kosticanja91@gmail.com; 4 Faculty of Sport, University UNION—Nikola Tesla, 11000 Belgrade, Serbia; aleksandar.pajic@mpn.gov.rs; 5 Department of Psychology of Assessment and Intervention, Heimerer College, 10000 Pristina, Kosovo; florim.gallopeni@kolegji-heimerer.eu

**Keywords:** kindergarten children motor ability, swimming ability, motor competence, preschool children, classification model

## Abstract

Swimming is a form of physical activity and a life-saving skill. However, only a few studies have identified swimming ability classifiers in preschool children. This pilot cross-sectional study aimed to find anthropometric (AM) and motor competence (MC) predictors of swimming ability in preschool children, by building classifiers of swimming ability group (SAG) membership. We recruited 92 children (girls n = 45) aged 5–6 years and took the AM and MC measurements in accordance with the reference manual and using the KTK battery test (motor quotient, MQ), respectively. A linear discriminant analysis tested a classification model of preschoolers’ swimming ability (SAG: POOR, GOOD, EXCELLENT) based on gender, age, AM, and MC variables and extracted one significant canonical discriminant function (model fit: 61.2%) that can differentiate (group centroids) POOR (−1.507), GOOD (0.032), and EXCELLENT (1.524). The MQ total was identified as a significant classifier, which absolutely contributed to the discriminant function that classifies children’s swimming ability as POOR (standardized canonical coefficient: 1.186), GOOD (1.363), or EXCELLENT (1.535) with an accuracy of 64.1%. Children with higher MQ total ought to be classified into higher SAG; thus, the classification model of SAG based on the MQ total is presented.

## 1. Introduction

Swimming ability, as a narrower term of aquatic competence [1], has been acknowledged a life-saving skill [2,3], since drowning is one of the most common causes of unintentional injury deaths throughout the world [4]. As the self-propulsion of a person through water, swimming is a physical activity used in sports performance, recreation, and therapy. Swimming ensures many health benefits across all ages, mostly represented in the current literature as an effective nonpharmacological intervention for children with asthma [5]. Previous studies have also proposed that swimming has a beneficial impact on pulmonary functions and respiratory muscle strength of healthy boys [6] and cardiorespiratory fitness and body composition of healthy children [7,8]. Moreover, swimming develops motor competence (MC) of healthy children [9,10]. On the contrary, if the water fails to meet water quality standards, exposure to disinfection byproducts in indoor swimming pool water may indicate numerous health problems (e.g., cough, eye irritation, and rash) [11,12]. Otherwise, the air inhaled above the surface of the water is clean and rich in oxygen.

Swimming ability requires the development of motor skills in the aquatic environment, although the current literature has been provided with the proofs about swimming reflex occurrence as an infantile reflex [13]. Basic motor skills in the aquatic environment (aquatic motor skills) are balance, breathing, propulsion, jumps, and manipulations [14]. According to Langendorfer and Bruya’s adaptation of Gallahue’s motor skills development model [15], the development of swimming ability in early childhood onsets with adaptation to the aquatic environment. The International Swimming Federation (FINA) [14] proposed a three-stage teaching method that allows for children’s progressive behavioral change as a result of the sequential learning of basic motor skills [16] while ensuring the following three underlying cornerstones: hierarchy, differentiation, and individualization of basic aquatic motor skills [15]. Therefore, the first stage establishes the fundamentals of adaptation to the aquatic environment by developing MC. MC’s primary focus is the learning of gross motor skills (adaptation to the place, flotation, displacement, immersion, passages, and jump) and fine motor skills (manipulations, spatial orientation, rhythm, kinaesthetic differentiation, and reaction) while aiming to develop basic aquatic motor skills at the second stage. Adaptation to the aquatic environment involves the acquisition of basic aquatic motor skills, which corresponds to the first two phases of the model, the reflex skills and aquatic readiness skills stages, according to Langendorfer and Bruya. Blanksby et al. suggested that the period between five and six years of age is optimal for children to learn the front crawl swimming stroke [17].

Evaluation of MC development and gross and fine motor skills in preschool children is limited to several frequently used assessments acknowledged in the current literature: Athletic Skills Track (AST), Democritos Movement Screening Tool for preschool children (DEMOST-PRE), Movement Assessment Battery for Children (MABC), Body Coordination Test for Children (KTK), Test of Gross Motor Development second edition (TGMD-2), and Motor-Proficiency-Test for children 4–6 year (MOT 4–6) [18]. Each of these battery tests uniquely assesses MC and is either a process- or product-oriented MC assessment (or both) [19]. KTK is a product-oriented assessment and is widely used to investigate the MC development in longitudinal designs because each test item is congruent across ages [20,21], as well as the MC development dependency on other physiological variables [22], and intervention-induced effects [23].

To the best of our knowledge, only three studies previously modeled children’s swimming ability—two in the function of sociodemographic characteristics [24,25], and one in the function of body size [26]. Previous studies have instead investigated the effects of swimming practice on MC [9,10]. Nonetheless, neither of them investigated the impact of MC on swimming performance nor swimming ability in preschool and school-age children and adolescents. However, several studies disclosed that higher levels of soccer- and karate-specific motor skills are related to higher levels of MC [27,28,29,30]. Moreover, preschool children who developed MC at higher levels were more frequently involved in physical activity, games, and sports, as compared to inactive peers [31]. The current literature yields that changes in preschool children’s MC is positively related to changes in time spent physically active [31,32,33] and levels of physical fitness [34,35,36]. Physical fitness levels follow increasing children’s physical activity levels [37] and essentially impact health [38]. Thus, developing and establishing positive health behaviors at an early age alter growth trajectories of health behaviors throughout development and into adulthood [32,39,40].

The physiological, biomechanical, and anthropometric predictors of swimming performance in young athletes have been broadly examined [29,41,42,43,44]. Nevertheless, to our knowledge, only one study investigated anthropometric (AM) predictors of swimming ability in preschool children [26]. They emphasized body weight as a predictor of swimming ability in preschool children. Among AM predictors of swimming performance in athletes are limb length and arm span [41,43]. From everything identified, few studies provide an in-depth analysis of AM measurements influence preschool children’s swimming ability in the current literature.

Therefore, this study aimed to find classifiers of swimming ability among the studied AM and MC variables in preschool children, thus building a predictive model that classifies children at the age of five years according to swimming ability group.

## 2. Materials and Methods

### 2.1. Participants

A total of 92 children (girls n = 45) at the age of five years (mean ± SD: 5.58 ± 0.5) were included in this pilot cross-sectional study. The inclusion criteria were that children were healthy preschoolers who had attended the ELC “English Language Centre” in Prishtina, Republic of Kosovo, who previously had familiarization and the first contact with the aquatic environment, and whose parents/guardians signed the written informed consent. The exclusion criteria were children who have any acute and/or chronic diseases (e.g., musculoskeletal injury, asthma), and who cannot swim at all (fear of water and cannot float without swimming float). The children’s parents/guardians were given written informed consent to read and sign, during which they were asked does a child have a fear of water and can float without any kind of swimming float. The study was carried out following the Declaration of Helsinki and approved by the ethics committee of the Faculty of Sport and Physical Education at the University of Novi Sad (ref. no. 21/2019).

### 2.2. Target Variable

#### Swimming Ability

Previous studies have evaluated swimming ability in children using the swimming ability scale (0–10-point scale) via a validated survey [24,25]. In this study, swimming coaches observed actual children’s swimming ability given the swimming ability scale, unlike in the previous studies where parents rated their children’s swimming ability without actual observation. Afterward, for research purposes, we labeled their swimming ability as POOR, GOOD, or EXCELLENT (Table 1).

The scale was slightly adapted for research purposes. The children were able to float without any kind of swimming float because all children previously had familiarization and first contact with the aquatic environment. Therefore, we did not include the lowest points (1 and 2). Furthermore, we excluded the highest points (8, 9, and 10) since no recruited children could swim more than one pool length.

### 2.3. Discriminating Variables

#### 2.3.1. Anthropometric (AM) Variables 

The anthropometric measurements were made in accordance with the reference manual of anthropometric standardization [45]. All kids were barefoot and minimum dressed and measured from the left side.

We measured body weight (SECA 804, Hamburg, Germany), height (SECA 214, Hamburg, Germany), and body mass index (weight(kg)/height(m^2^)). Arm span, shoulder width, lengths (foot, trunk, hand, and arm), diameters (biacromial and bicristal), and breadths (elbow, knee, and ankle) were measured using the Martin anthropometer (GPM Anthropometer 100; DKSH Switzerland Ltd., Zurich, Switzerland). Circumferences (chest, abdominal, and thigh) were measured with tape adhered to the skin. Skinfolds (abdomen, thigh, and calf) were measured with a Lange skinfold caliper (Cambridge Scientific Instruments, Cambridge, MD), and the results were read 3 s after placing the caliper. The results for all anthropometric measurements were registered and recorded when two consecutive measurements have coincided inside 0.1 cm, except for body weight and skinfolds, which must have coincided with 0.1 kg and 4 mm, respectively.

#### 2.3.2. Motor Competence (MC) Variables

The construct of MC [46], gross motor skills (locomotor, stability and manipulative skills) and fine motor skills, has been referred to the fundamental movement skills in this study because preschool children are studied population [16]. We evaluated children’s MC using the Körper-Koordinationtest für Kinder (KTK) battery test, a non-sport/skill-specific test consisting of the following four test items: balance beam, single-lever jumps, lateral jumps, and transfers on platforms [47]. Two gross motor skills components are assessed using the KTK—locomotor and stability skills. The raw performance score of each test item was converted into a standardized motor quotient (MQ), adjusted for age and gender according to normative data tables based on the performance of a standardization sample. In the same manner, the sum of all four item MQs was transformed into MQ total. KTK has been previously recognized as a reliable evaluation method of MC development in preschoolers (ICC = 0.89) [48], and hence, it has been used for the criterion validity studies of other MC evaluation method [49].

##### Balance Beam Task

We asked the children to walk backward on three balance beams. The beams’ dimensions are 3 m in length, 5 cm in height, but with decreasing widths of 6, 4.5, and 3 cm. Children had three trails at each beam, and the number of successful steps was recorded; a maximum of 24 steps (eight per trial) was counted for each balance beam (maximum of 72 steps).

##### Single-Lever Jumps Task

Children were instructed to hop on one foot at the time, preceding the jump over a stack of foam blocks after a short run-up. After a successful hop with each foot, the height was increased by adding new blocks (50 cm, 20 cm, 5 cm). A successful hop indicated that a child jumped over the block without touching it and successfully continued hopping on the same foot at least two times. A child had three trails at each height and on each foot; three, two, or one point(s) were awarded for successful completion on the first, second, or third trial, respectively; a maximum of 39 points (12 stacks of blocks) could be awarded for each leg (maximum score of 78).

##### Jumping Sideways Task

Children were asked to make consecutive jumps, with both feet together, from side to side over a beam (60 × 4 × 2cm) as quickly as possible in 15 s. The number of correct jumps in two trials was summed and recorded.

##### Moving Sideways Task

The child began a trial by standing with both feet on one platform (25 × 25 × 2cm) supported on four legs, 3.7 cm in height. The subject held the second identical platform in his/her hands; then, the children were instructed to place the second platform alongside the first one and, then, to step onto it; the first platform was then lifted and placed alongside the second, and the child stepped onto it. Two points were awarded for each successful transfer from one platform to another (one point for shifting the platform, the other for transferring the body onto the second one). If the child fell off the platform in the process, he/she stepped back onto the platform and continued the test. The total number of points for the 20 s from two trials was recorded.

### 2.4. Procedures

The testing procedures were carried out in January–February 2020 at two locations, in the lighted gym (anthropometric measurements and MC evaluation) and indoor swimming pool in Prishtina, Republic of Kosovo. Three swimming coaches, licensed by the Federation of Water Sports of Kosovo, observed how children navigated in water according to the swimming ability scale (item-by-item) and recorded it accordingly. The agreement among the raters has been excellent (ICC = 0.81). The same experienced examiner, a research assistant, took all AM measures of all children. Two experienced examiners, also research assistants, evaluated children’s MC using the KTK, and the agreement among them was excellent (ICC = 0.92).

We attempted to build a classification model of preschoolers’ swimming ability (target variable: swimming ability group SAG—POOR, GOOD, and EXCELLENT) based on the following discriminating variables: gender, age, AM variables (body weight, body height, Body mass index, arm span, shoulder width, foot length, trunk length, hand length, arm length, chest circumference, abdominal circumference, thigh circumference, biacromial diameter, bicristal diameter, elbow breadth, knee breadth, ankle breadth, abdomen skinfold, thigh skinfold, and calf skinfold), and MC variables (MQs for balance beam, single-lever jumps, jumping sideways, moving sideways, and total).

### 2.5. Statistical Analysis

Data were analyzed using SPSS v. 20.0 (IBM SPSS, Inc., Chicago, IL, USA) and presented as mean (SEM) unless otherwise stated. A Shapiro–Wilk test failed to reject normality, and the Levene’s and Box’s M tests indicated equal variance-covariance matrices within the groups. Before testing the primary study hypothesis, a T-test for independent samples (T-test) was used to evaluate the gender effect on discriminating variables.

We used a linear discriminant analysis (LDA) with equal prior probabilities to build classifiers of SAG membership. First, to identify potential classifiers of SAG before building a preschoolers’ swimming ability classification model, the age, gender AM and MC variables, and SAG dependency were tested. A Mann–Whitney U tests estimated the gender and age effects on SAG, separately, and a one-way ANOVA with Bonferroni correction for multiple comparisons tested whether the AM and MC variables on average differ across the SAG. For ANOVA models, eta squared (ŋ^2^) is reported as a measure of effect size, and defined as small (ŋ^2^ = 0.01), medium (ŋ^2^ = 0.06), and large (ŋ^2^ = 0.14) [18]. Additionally, the correlation matrix of pairwise Pearson’s correlation coefficient (r) was created to assess multicollinearity (r > 0.7) between potential classifiers [50].

Finally, we modeled the difference between the SAG by finding the linear combination of the independent variables. A predictive model of SAG membership included independent variables that found to be significantly affected by the main effect of SAG, according to the one-way ANOVA and Mann–Whitney U tests, and a stepwise method selected classifiers from the entered independent variables. Wilks’ Lambda (Wilks’ λ) tested variable contribution to a discriminant function (Exact F test), and whether the discriminant function explains the group membership well (Chi-Square (χ²)). An LDA created one canonical discriminant function to maximize the difference in mean discriminant score between POOR, GOOD, and EXCELLENT SAG (centroids). Thus, the canonical discriminant function was constructed in the following equation:

(1) $$D_{j}=c+c_{1}X_{1j}$$

where D_j_ is discriminant score for j^th^ observation, c are unstandardized canonical coefficients, and X_1j_ is the j^th^ observation for the first variable.

There was a linear discriminant function (LDF) defined for the model that classifies kids’ swimming skills as POOR, GOOD, EXCELLENT in the following form:

(2) $$C_{k}=c_{k}+c_{1}X_{1j}$$

where C_k_ is classification score (classification function value) for group k, c are standardized canonical coefficients of LDF, and X_1j_ is the j^th^ observation for the first variable.

We reported model fit information (eigenvalue λ, square of canonical correlation coefficient Rc^2^), and model’s probability of detection (true positive rate; TPR) and probability of false alarm (true negative rate; TNR). Press’s Q statistic was additionally computed to compare the discriminatory power of the classification with the classification accuracy expected by chance. The cross-validation estimated the classification performance of the predictive model. Level of significance was set a prior at *p* ≤ 0.05.

## 3. Results

### 3.1. Sample Characteristics

All children completed the testing procedures. The location and dispersion of characteristics for total and stratified sample by gender are presented in Table 2. The T-test showed that boys had significantly higher mean of diameter and breadth measures, and chest circumference, shoulder width, arm span, and length compared to girls. On average, boys also were significantly taller than girls (Table 2).

### 3.2. LDA of Swimming Abiliy and the AM and MC Variables

#### 3.2.1. SAG Characteristics

The children were assigned to one of three SAGs (POOR: girls n = 14, boys n = 17, age = 5.71 ± 0.08; GOOD: girls n = 17, boys n = 14, age = 5.55 ± 0.09; and EXCELLENT: girls n = 14; boys n = 16, age = 5.47 ± 0.09).

A Mann–Whitney U test did not show significant difference in SAG mean ranks between genders (boys vs. girls: 46.17 vs. 46.84; Z = −0.128, *p* = 0.898) and across ages (5 vs. 6: 52.36 vs. 42.19; Z = −1.915, *p* = 0.056).

#### 3.2.2. Difference in the AM and MC Variables across the SAG

Table 3 presents the main effect of SAG from a one-way ANOVA model. There has not been shown a significant main effect of SAG on the studied AM variables, and, thus, the children in POOR, GOOD, and EXCELLENT SAG on average did not differ in the studied AM variables.

A one-way ANOVA, however, demonstrated a large significant main effect of SAG on all MC variables (balance beam: ŋ^2^ = 0.269; single-lever jumps: ŋ^2^ = 0.313; jumping sideways: ŋ^2^ = 0.518; moving sideways: ŋ^2^ = 0.612), and a Bonferroni post-hoc test revealed that the mean MQ for all test items significantly differed across SAG, except for the balance beam and moving sideways. As compared to GOOD and EXCELLENT, POOR had significantly lower mean MQ for total (*p* < 0.001) and all test items (*p* < 0.001), except for balance beam, in which POOR and GOOD did not differ (*p* = 1.0). We observed a significantly higher mean MQs of EXCELLENT, comparing to the remaining groups, for total (*p* < 0.001) and all test items (*p* < 0.001), except for moving sideways, where GOOD and EXCELLENT did not differ (*p* = 0.157). Table 3 presents detailed information on the results from the analysis.

#### 3.2.3. Correlation Matrix of Pearson’s r for Discriminating Variables

Correlation matrix of Pearson’s r was created only for MC variables (Table 4) because a one-way ANOVA demonstrated a significant main effect of SAG only on the MC variables, and a Mann–Whitney U test did not show significant difference between genders and across ages in SAG. Pearson’s r indicates that only MQ Total and each MQ test item tend to be significantly and positively correlated (r from 0.618 to 0.844, *p* < 0.05), and significant, but lower positive correlations were observed between the each MQ test item (r from 0.126 to 0.706, *p* < 0.05), excluding MQ Total.

#### 3.2.4. Model Specification

The AM variables, gender, and age were not included in a predictive model of SAG membership, because a one-way ANOVA did not demonstrate a significant main effect of SAG on AM variables, and a Mann–Whitney U test did not show significant difference between boys and girls, and across ages in the SAG. Therefore, we tested a predictive model of SAG membership based on all MQs using a stepwise LDA. Box’s M test indicated equal population covariance matrices within groups (Box’s M = 0.429, F_(2, 17812.01)_ = 0.211, *p* = 0.810). A LDA created one canonical discriminant function in the first step, that differentiates scores among the groups significantly (model fit: λ = 1.574, Rc^2^ = 0.612, χ²(2) = 84.161, *p* < 0.0005), and explains the SAG membership well (Wilk’s λ_(1, 2, 89)_ = 0.388). Standardized canonical discriminant function coefficient implied that only MQ total (1.0) among studied discriminating variables has ability to predict SAG. One canonical discriminant function was created, because the MQ total was extracted as a single significant contributor to the canonical discriminant function (Exact F_(2, 89)_ = 70.063, *p* < 0.0005). MQs for balance beam (Wilk’s λ_(1, 2, 89)_ = 0.731), single-lever jumps (Wilk’s λ_(1, 2, 89)_ = 0.687), jumping sideways (Wilk’s λ_(1, 2, 89)_ = 0.482), and moving sideways (Wilk’s λ_(1, 2, 89)_ = 0.635) contributed less to the canonical discriminant function, in comparison with MQ total (Wilk’s λ_(1, 2, 89)_ = 0.388). Although the canonical structure matrix showed correlations above 0.3 (Table 5), the MQ total had the highest discriminant loading on discriminant function, and thus, we assigned a label to the discriminant function accordingly. The following canonical discriminant function was used to compute discriminant score (D_j_) for each subject:

(3) $$D_{j}=-11.801+0.115\times MQTotal$$

The lowest centroid was observed in POOR (group centroid = −1.507) as compared to GOOD (group centroid = 0.032), and EXCELLENT (group centroid = 1.524).

#### 3.2.5. Classification Model of SAG Membership

Finally, we defined three LDFs (Equation (2)) for the model to classify kids’ swimming ability as POOR, GOOD, or EXCELLENT based on their MQ total, by computing subject’s classification score (C_k_) for each group (Table 6), and assigning children to a SAG for which the classification score was the highest.

LDA correctly classified 65.2% of original grouped cases. The model’s probability of detection of children with EXCELLENT SAG is the highest (TPR = 76.7%) as compared to POOR SAG (TPR = 71.0%), and GOOD SAG (TRP = 48.4%), while probability of false alarm is the highest for GOOD SAG (TNR = 51.6%). POOR (TNR = 32.2%) and EXCELLENT SAG (TNR = 19.4%) tend to have lower probabilities of false alarm as compared to GOOD SAG (Table 7). According to Press’s Q statistic, the predictive model of SAG membership based on MQ total exceeds the classification accuracy expected by chance at a statistically significant level (Press’s Q = 42.09 > critical value for χ²_(1)_ = 6.63). Model cross-validation indicates an accuracy of 64.1%.

## 4. Discussion

This is the first study on identifying AM and MC predictors of swimming ability in preschool children by building classifiers of SAG membership. Our preliminary results indicate that swimming ability does not depend upon age, gender, and AM variables in five-year-old children. Hence, LDA defined a linear classification model of SAG (model fit: λ = 1.574, χ²_(2)_ *=* 84.161, *p* < 0.0005) solely based on MQ total, that classifies kids into SAG with an accuracy of 64.1% (cross-validation). The proposed linear classification model explains 61.2% of the variance in the SAG, and the SAG membership well (Wilk’s λ_(1, 2, 89)_ = 0.388). Three LDFs were defined for classification score computation for POOR (intercept = −54.080, standardized canonical coefficient = 1.186), GOOD (intercept = −71.100, standardized canonical coefficient = 1.363), and EXCELLENT (intercept = −89.876, standardized canonical coefficient = 1.535) SAG. The most likely detected children were those for the EXCELLENT (TRP = 76.7%) and POOR (TRP = 71%), while the children for the GOOD were most likely misclassified (TNR = 51.6%), as compared to the remaining classes.

To the best of our knowledge, only three studies have previously modeled the swimming ability in children [24,25,26]. Pharr et al. identified several biological (race, gender, age) and socioeconomic and environmental predictors. Significant socioeconomic and environmental predictors are (parental swimming ability, parent encourages a child to swim, best friend enjoys swimming, the pool opened all year, a child knows how to be safe around water, and fear of drowning). In preschool children, these predictors explained 53% of the total swimming ability variance [24]. Both studies that examined demographic SAG predictors observed significantly higher swimming ability levels in older children and adolescents. Also, male children and adolescents, compared to younger and female peers, respectively [24,25]. Our findings, however, suggest that swimming ability did not significantly depend upon the gender (*p* = 0.898) and age (*p* = 0.056), although five-year-old kids tended to have higher mean SAG rank comparing to 6-years-old kids, and previous studies showed that children’ swimming ability significantly depends on age [24,25]. However, the lack of a significant age effect on SAG may be attributed to the small size of the subsample of subjects and short age-interval (5–6 years old). Previous studies observed a significant age effect on swimming ability in the broader age interval (4–11 years old). To the authors’ knowledge, previous studies have mostly addressed gender and age effects on swimming performance in young athletes [29,42,43] but rarely on swimming ability among children.

The third study examined the bodyweight influence on swimming ability in children and found it a significant predictor of swimming ability [26]. Otherwise, the impact of anthropometric measurements in children on swimming ability has not yet been broadly investigated. Still, preceding studies demonstrated that swimming performance significantly depends on AM variables in young athletes [42], especially on arm span [41,43]. Our study, however, emphasized no significant differences between kids in POOR, GOOD, and EXCELLENT SAG in anthropometric measurements. The mean body height and weight, BMI, shoulder width, lengths (foot, trunk, arm, and hand), circumferences (chest, abdominal, and thigh), diameters (biacromial, bicristal), breadths (elbow, knee, ankle), and skinfolds thickness (abdomen, thigh, and calf) were most likely similar across the SAG. On average, POOR, GOOD, and EXCELLENT SAG also had, but less likely, similar arm span (*p* = 0.175) as compared to those above. Therefore, it can be assumed that at an early stage of swimming skill development, the anthropometric measurements do not play a significant role and that their contribution grows with long-term swimming performance development. We can also theorize that the small size of the subsamples (n≈30) could have contributed to a non-significant difference occurrence in arm span if the effect of SAG was small. Hitherto, examining the real relationship between swimming ability and AM variables among children, remains the focus of future studies. Additionally, providing a representative sample of subjects and reliable diagnostic tools to assess body composition (e.g., DEXA) and swimming ability (e.g., 3D human motion analysis).

There has been a lack of researches about MC effects on the swimming performance and swimming ability of preschool children in the current literature. However, previous studies investigated the effects of swimming practice on MC [9,10]. Hence, this is the first study that relates MC to swimming ability and evaluates its contribution to preschool children’s swimming ability. Our study highlighted the MQ total as a classifier that contributes the most to the canonical discriminant function comparing to each MQ test item, given the correlation canonical structure matrix. The MQ total was selected as a classifier of SAG using a stepwise method, and withal MQ total integrates MQs for all test items, as well as gender and age. Selecting MQ total overall four test items as a classifier of SAG may also have benefit, because, utilizing the correlation matrix of MC variables, the observed correlation between MQs for jumping and moving sideways indicates multicollinearity.

This pilot study indicates that the MQ total, as it has solely contributed to the canonical discriminant function, can differentiate (group centroid) POOR (−1.507), GOOD (0.032), and EXCELLENT (1.524) and that children with higher MC total levels ought to have higher levels of swimming ability (standardized canonical coefficients: POOR 1.186; GOOD 1.363; and EXCELLENT 1.535). This is in line with the previous studies which reported that higher MC levels were related to higher levels of other sport-specific motor skills in beginner karatekas [30] and youth soccer players [27,28].

Early childhood is highlighted as a critical period for development of MC (fundamental movement skills) [13], and thus, it is congruent to the adaptation to aquatic environment [14]. Adaptation to the aquatic environment depends on the acquisition of basic gross and fine motor skills, whose acquisition will, in turn, ensure MC foundation, which will allow for the acquisition of basic and then specific aquatic motor skills as a result of sequential learning [14,16]. Therefore, significantly fewer swimming lessons were optimal to learn basic front crawl strokes at the age of five compared to 2, 3, and 4-year-old peers [17]. Hence, MC, which refers to fundamental movement skills here, should be developed a priori.

In agreement with those mentioned above, the practice of various sports activities resulted in a greater increase of physical fitness compared to single-sport activity in preschool and school-age children [51,52]. Also, preschool children who practiced single-sport activity developed MC more than inactive peers [9]. Webster et al. disclosed that engagement in vigorous physical activity has the highest impact on the MC development in preschool children [53].

Numerous factors influence MC development besides behavioral factors (sedentary time, intensity-specific physical activity, and type of organized sports activities), and, hence, mediate MC effect on the SAG. Niemistö et al. [54] found that the MC levels (model fit = 38%) assessed using the KTK in preschool children significantly depend on biological factors (biological maturation, gender, and temperament traits), participation in organized sports, as well as parents’ education level. We did not find gender as a significant predictor of the MQ variables, although their study showed a significantly higher mean MC in boys and higher mean score for balance test item in girls. Another study showed that the residential density was negatively associated with MC levels assessed by TGMD-3 [55].

Previous studies reported that higher MC levels, especially higher levels of locomotor skills, are positively related to time spent physically active and enjoyment in physical activity during childhood and adolescence, while positively affecting health [32,33,39,40]. Therefore, besides being necessary for the learning of aquatic motor skills, the emerging role of age-appropriate MC development of preschool children is also in establishing long-term physical activity and motor skill performance.

This classification model may be introduced to kindergarten teachers and coaches who could assess children’s MC using the KTK and predict if a child is ready to start to learn to swim by predicting how will be a child’s swimming ability rated. This would ensure optimal MC basis for acquiring basic and later, specific, aquatic motor skills, which would result in a shorter duration of the learning process. Moreover, this confirms that swimming coaches should also incorporate non-specific MC development in early childhood teaching programs.

We evaded exploring the relationship between swimming ability and manipulative skills and fine motor skills, although we inspected the relationships between swimming ability and stability and locomotor skills. Therefore, future studies should provide an in-depth analysis of MC relative to swimming ability, while encompassing the whole MC construct by evaluating actual MC using available battery tests that are both, process- and product-oriented. Moreover, the replicability of the swimming ability scale and this classification model should be further examined. Finally, to build a better-fitted model of SAG membership, preceding findings of sociodemographic and environmental contributions to swimming ability should also be incorporated in a swimming ability classification model. Ultimately, the mediating effects of behavioral, biological, and sociodemographic factors which influence MC development should be embraced as well. Therefore, future studies are obliged.

## 5. Conclusions

These preliminary results indicate that the KTK battery test can differentiate children’s swimming ability at the age of five years and that children with higher MQ ought to be classified into higher levels of SAG. Thus, a model has been built to predict into which SAG children will be classified. Our pilot study, however, did not identify age, gender, and AM variables as the significant classifiers of SAG membership. Therefore, the MC development should be primary in focus in early childhood, which will allow mastering gross and fine motor skills at an age-appropriate level. This approach will consequently provide optimal MC foundation for learning of specific motor skills in preschool children.

## Figures and Tables

**Table 1:** Swimming ability scale by grades.

Point	Item	SAG
0	I avoid getting near/in water except to bathe	Excluded from the study
1	Cannot swim at all	
2	Can splash around, shallow end	POOR
3	Can put face in water, blow bubbles	
4	Can hold head under water (5–10 s)	
5	Can glide a little, face in water, shallow end only	GOOD
6	Can swim a little in the deep end; face in water; can float a little	
7	Can swim with a true front crawl stroke, 1 pool length, no stooping	EXCELLENT
8	Can swim front crawl stroke, 2 or 3 pool lengths; can tread water for 5–10 min	Excluded from the study
9	Can swim 4 or more pool lengths, no stooping; knows 2 or 4 different strokes.	
10	Can swim many lengths without stopping; on a swim team or could be on a swim team	

**Table 2:** Characteristics for the total sample and stratified by gender.

	Total	Boys (N = 47)	Girls (N = 45)
Age	5.58 ± 0.50	5.55 ± 0.57	5.60 ± 0.48
Body weight (kg)	23.62 ± 4.31	24.49 ± 4.91	22.72 ± 3.40
Body height (cm)	118.32 ± 5.48	119.33 ± 6.00 *	117.25 ± 4.70
BMI (kg/m^2^)	16.84 ± 2.22	17.20 ± 2.50	16.46 ± 1.84
Arm span (cm)	116.97 ± 6.27	118.29 ± 6.55 *	115.59 ± 5.71
Shoulder width (cm)	50.38 ± 2.90	51.01 ± 3.04 *	49.74 ± 2.62
Foot length (cm)	58.96 ± 3.75	58.80 ± 4.30	59.12 ± 3.13
Trunk length (cm)	65.79 ± 3.68	66.50 ± 4.42	65.04 ± 2.56
Hand length (cm)	18.85 ± 1.10	19.01 ± 1.18	18.68 ± 1.00
Arm length (cm)	13.01 ± 0.83	13.21 ± 0.84 *	12.80 ± 0.77
Chest circumference (cm)	59.05 ± 4.68	60.22 ± 4.74 *	57.84 ± 4.32
Abdominal circumference (cm)	54.98 ± 6.13	55.86 ± 6.79	54.06 ± 5.28
Thigh circumference (cm)	34.13 ± 3.47	33.87 ± 3.84	34.41 ± 3.04
Biacromial diameter (cm)	26.83 ± 1.65	27.25 ± 1.76 *	26.39 ± 1.40
Bicristal diameter (cm)	19.74 ± 1.44	20.04 ± 1.54 *	19.44 ± 1.27
Elbow breadth (cm)	4.83 ± 0.34	4.94 ± 0.36 **	4.72 ± 0.28
Knee breadth (cm)	7.13 ± 0.50	7.32 ± 0.53 **	6.94 ± 0.39
Ankle breadth (cm)	5.15 ± 0.48	5.31 ± 0.52 **	4.98 ± 0.38
Abdomen skinfold (cm)	5.80 ± 3.08	5.45 ± 2.90	6.18 ± 3.25
Thigh skinfold (cm)	14.97 ± 6.10	14.68 ± 6.67	15.27 ± 5.51
Calf skinfold (cm)	14.48 ± 4.96	13.94 ± 5.25	15.04 ± 4.63
Balance beam MQ	102.79 ± 12.69	101.45 ± 13.49	104.20 ± 11.78
Single-lever jumps MQ	120.29 ± 18.74	123.96 ± 17.23	116.47 ± 19.67
Jumping sideways MQ	80.30 ± 10.69	80.53 ± 11.45	80.07 ± 9.96
Moving sideways MQ	103.50 ± 15.00	101.66 ± 15.35	105.42 ± 14.54
MQ Total	102.43 ± 13.77	102.30 ± 13.71	102.58 ± 13.99

Values are mean ± SEM; Abbreviations: BMI—body mass index; MQ—Körper-Koordinationtest für Kinder (KTK) motor quotient; ** genders significantly different at *p* < 0.01; * genders significantly different at *p* < 0.05.

**Table 3:** Difference in the anthropometric (AM) and motor competence (MC) variables across the swimming ability group (SAG).

Outcomes	Poor (N = 31)	Good (N = 31)	Excellent (N = 30)	ANOVA F_2, 89_
Body weight (kg)	23.30 (0.92)	23.69 (0.72)	23.89 (0.68)	0.15, *p* = 0.864, ŋ^2^ = 0.003
Body height (cm)	117.95 (1.31)	118.52 (0.73)	118.49 (0.84)	0.10, *p* = 0.902, ŋ^2^ = 0.002
BMI (kg/m^2^)	16.78 (0.45)	16.80 (0.41)	16.93 (0.34)	0.04, *p* = 0.965, ŋ^2^ = 0.001
Arm span (cm)	115.42 (1.36)	117.11 (0.89)	118.42 (1.07)	1.78, *p* = 0.175, ŋ^2^ = 0.038
Shoulder width (cm)	49.75 (0.67)	50.58 (0.41)	50.84 (0.44)	1.19, *p* = 0.310, ŋ^2^ = 0.026
Foot length (cm)	58.64 (0.84)	58.95 (0.67)	59.30 (0.48)	0.23, *p* = 0.796, ŋ^2^ = 0.005
Trunk length (cm)	66.15 (0.95)	65.50 (0.41)	65.71 (0.51)	0.25, *p* = 0.779, ŋ^2^ = 0.006
Hand length (cm)	18.73 (0.25)	18.93 (0.17)	18.89 (0.17)	0.27, *p* = 0.765, ŋ^2^ = 0.006
Arm length (cm)	12.84 (0.16)	13.12 (0.14)	13.07 (0.15)	1.02, *p* = 0.366, ŋ^2^ = 0.022
Chest circumference (cm)	59.04 (0.95)	58.45 (0.75)	59.70 (0.83)	0.54, *p* = 0.583, ŋ^2^ = 0.012
Abdominal circumference (cm)	55.03 (1.23)	54.88 (1.16)	55.04 (0.94)	0.01, *p* = 0.993, ŋ^2^ = 0.000
Thigh circumference (cm)	33.89 (0.62)	34.17 (0.65)	34.35 (0.62)	0.13, *p* = 0.878, ŋ^2^ = 0.003
Biacromial diameter (cm)	6.86 (0.36)	26.51 (0.25)	27.13 (0.27)	1.11, *p* = 0.335, ŋ^2^ = 0.024
Bicristal diameter (cm)	19.72 (0.29)	19.72 (0.23)	19.80 (0.26)	0.03, *p* = 0.967, ŋ^2^ = 0.001
Elbow breadth (cm)	4.81 (0.07)	4.76 (0.06)	4.93 (0.05)	2.11, *p* = 0.126, ŋ^2^ = 0.045
Knee breadth (cm)	7.09 (0.10)	7.15 (0.10)	7.16 (0.06)	0.15, *p* = 0.861, ŋ^2^ = 0.003
Ankle breadth (cm)	5.12 (0.11)	5.15 (0.08)	5.19 (0.07)	0.15, *p* = 0.865, ŋ^2^ = 0.003
Abdomen skinfold (cm)	5.65 (0.46)	6.35 (0.69)	5.40 (0.50)	0.79, *p* = 0.457, ŋ^2^ = 0.017
Thigh skinfold (cm)	14.13 (1.16)	15.23 (1.07)	15.57 (1.10)	0.46, *p* = 0.633, ŋ^2^ = 0.010
Calf skinfold (cm)	14.35 (0.92)	14.03 (0.97)	15.07 (0.80)	0.34, *p* = 0.712, ŋ^2^ = 0.008
Balance beam MQ	96.97 (1.59)	99.65 (1.87)	112.07 (2.42)*	16.37, *p* < 0.001, ŋ^2^ = 0.269
Single-lever jumps MQ	107.29 (3.19)	121.13 (3.20) ^¥^	132.87 (1.86)	20.27, *p* < 0.001, ŋ^2^ = 0.313
Jumping sideways MQ	71.81 (1.32)	78.97 (1.33) ^¥^	90.47 (1.41)	47.84, *p* < 0.001, ŋ^2^ = 0.518
Moving sideways MQ	91.35 (2.15) *	106.68 (2.35)	112.77 (2.03)	25.52, *p* < 0.001, ŋ^2^ = 0.365
MQ Total	89.35 (1.66)	102.71 (1.51) ^¥^	115.67 (1.52)	70.06, *p* < 0.001, ŋ^2^ = 0.612

Values are mean (SEM); Abbreviations: BMI—body mass index; MQ—KTK motor quotient; ŋ^2^ eta squared; * a group significantly different from the remaining groups at Bonferroni adjusted *p* < 0.05; ^¥^ all groups significantly different at Bonferroni adjusted *p* < 0.05.

**Table 4:** The correlation matrix showing Pearson’s r for MQ variables.

	Balance Beam MQ	Single-Lever Jumps MQ	Jumping Sideways MQ	Moving Sideways MQ	MQ Total
Balance beam MQ		0.126	0.460 *	0.438 **	0.618 **
Single-lever jumps MQ			0.542 *	0.338 **	0.700 **
Jumping sideways MQ				0.706 **	0.844 **
Moving sideways MQ					0.766 **
MQ Total					

Abbreviation: MQ—KTK motor quotient; * significantly correlated at *p* < 0.05; ** significantly correlated at *p* < 0.01.

**Table 5:** The canonical structure matrix.

Motor Coordination Tests	MQ Function
Balance beam MQ ^e^	0.449
Single-lever jumps MQ ^e^	0.509
Moving sideways MQ ^e^	0.617
Jumping sideways MQ ^e^	0.663
MQ Total	1.000

Values are discriminant loadings. Abbreviation: MQ—KTK motor quotient; ^e^ Variable excluded from the analysis.

**Table 6:** Linear discriminant functions.

Computation of Classification Score for:	Model
POOR SSLbG	$C_{k}=-54.080+1.186\times MQTotal$
GOOD SSLbGs	$C_{k}=-71.100+1.363\times MQTotal$
EXCELLENT SSLbGs	$C_{k}=-89.876+1.535\times MQTotal$

Abbreviation: MQ—KTK motor quotient.

**Table 7:** Classification table.

	SAG	Predicted Group Membership			Total
		POOR	GOOD	EXCELLENT	
Count	POOR	22	9	0	31
	GOOD	10	15	6	31
	EXCELLENT	0	7	23	30
%	POOR	71.0	29.0	0.0	100.0
	GOOD	32.3	48.4	19.4	100.0
	EXCELLENT	0.0	23.3	76.7	100.0
**Cross-Validated**					
Count	POOR	21	10	0	31
	GOOD	10	15	6	31
	EXCELLENT	0	7	23	30
%	POOR	67.7	32.3	0.0	100.0
	GOOD	32.3	48.4	19.4	100.0
	EXCELLENT	0.0	23.3	76.7	100.0
